# Cloacal microbiota are biogeographically structured in larks from desert, tropical and temperate areas

**DOI:** 10.1186/s12866-023-02768-2

**Published:** 2023-02-11

**Authors:** H. Pieter J. van Veelen, Juan Diego Ibáñez-Álamo, Nicholas P. C. Horrocks, Arne Hegemann, Henry K. Ndithia, Mohammed Shobrak, B. Irene Tieleman

**Affiliations:** 1 Groningen Institute for Evolutionary Life Sciences, University of Groningen, Groningen, The Netherlands; 2 Wetsus, European Centre of Excellence for Sustainable Water Technology, Oostergoweg 9, 8911 MA Leeuwarden, The Netherlands; 3 Department of Zoology, Faculty of Sciences, University of Granada, Granada, Spain; 4 Cambridge Institute of Therapeutic Immunology & Infectious Disease (CITIID), Cambridge Biomedical Campus, University of Cambridge, Jeffrey Cheah Biomedical Centre, Puddicombe Way, Cambridge, CB2 0AW UK; 5 Department of Biology, Lund University, Ecology Building, 223 62 Lund, Sweden; 6 Ornithology Section, Department of Zoology, National Museums of Kenya, Nairobi, Kenya; 7 Biology Department, Science College, Taif University, P.O. Box 11099, Taif, 21944 Saudi Arabia; 8 National Centre for Wildlife, P.O. Box 61681, Riyadh, 11575 Saudi Arabia

**Keywords:** Alaudidae, Avian microbiota, Cloacal microbiota, Host-microbiome, Microbial biogeography

## Abstract

**Background:**

In contrast with macroorganisms, that show well-documented biogeographical patterns in distribution associated with local adaptation of physiology, behavior and life history, strong biogeographical patterns have not been found for microorganisms, raising questions about what determines their biogeography. Thus far, large-scale biogeographical studies have focused on free-living microbes, paying little attention to host-associated microbes, which play essential roles in physiology, behavior and life history of their hosts. Investigating cloacal gut microbiota of closely-related, ecologically similar free-living songbird species (*Alaudidae,* larks) inhabiting desert, temperate and tropical regions, we explored influences of geographical location and host species on α-diversity, co-occurrence of amplicon sequence variants (ASVs) and genera, differentially abundant and dominant bacterial taxa, and community composition. We also investigated how geographical distance explained differences in gut microbial community composition among larks.

**Results:**

Geographic location did not explain variation in richness and Shannon diversity of cloacal microbiota in larks. Out of 3798 ASVs and 799 bacterial genera identified, 17 ASVs (< 0.5%) and 43 genera (5%) were shared by larks from all locations. Desert larks held fewer unique ASVs (25%) than temperate zone (31%) and tropical larks (34%). Five out of 33 detected bacterial phyla dominated lark cloacal gut microbiomes. In tropical larks three bacterial classes were overrepresented. Highlighting the distinctiveness of desert lark microbiota, the relative abundances of 52 ASVs differed among locations, which classified within three dominant and 11 low-abundance phyla. Clear and significant phylogenetic clustering in cloacal microbiota community composition (unweighted UniFrac) showed segregation with geography and host species, where microbiota of desert larks were distinct from those of tropical and temperate regions. Geographic distance was nonlinearly associated with pairwise unweighted UniFrac distances.

**Conclusions:**

We conclude that host-associated microbiota are geographically structured in a group of widespread but closely-related bird species, following large-scale macro-ecological patterns and contrasting with previous findings for free-living microbes. Future work should further explore if and to what extent geographic variation in host-associated microbiota can be explained as result of co-evolution between gut microbes and host adaptive traits, and if and how acquisition from the environmental pool of bacteria contributes to explaining host-associated communities.

**Supplementary Information:**

The online version contains supplementary material available at 10.1186/s12866-023-02768-2.

## Background

For animals and plants, strong and consistent biogeographical patterns of distribution exist and are associated with local adaptation of physiology and life-history traits [1–4]. In contrast, for microbes such a consistency in large-scale biogeographical patterns has not been found (e.g. [5–7], fueling a debate about the ecological and evolutionary processes that govern spatial variation in different life forms [8–10]. Well-established patterns in plants and animals like the greater diversity towards the tropics or the decay of community similarity with geographic distance are often not detected in free-living microbes ([5, 6, 7, 11], but see [12]). Several reasons have been proposed to explain this discrepancy, including differences in the spatial scales at which dispersal ability and environmental selection affect microbes, compared with plants/animals. Also, differences in taxonomic levels of analysis between macro (e.g. species) and microorganisms (e.g. Amplicon Sequence Variants – ASVs), and other methodological issues can play a role such as inability to differentiate inactive/dead microorganisms, or underestimation of microbial diversity [9, 10]. Earlier studies with free-living microbes supported Baas-Becking’s paradigm that the local environmental conditions can select and maintain distinctive microbial assemblages [10, 13], while the current debate concentrates on whether “everything is everywhere”, and on the microbial traits that determine the geographical distribution of microorganisms [8, 10]. However, thus far, microbial biogeography has mainly focused on free-living microbial assemblages in aquatic or terrestrial environments, paying little attention to host-associated microbes [14] despite the ubiquitous occurrence of host-microbe associations in nature [15].

In the context of understanding biogeographical patterns and adaptations, host-associated microbes present an especially interesting case. The environment that host-associated microbes inhabit is the host’s body, which—for many host taxa including birds and mammals—is generally relatively constant in terms of factors such as pH, temperature and salinity, providing a similar environment for host-associated microbes across different biogeographical areas and despite large geographical distances. Dispersal of host-associated microbes is not well-understood and may differ from dispersal of environmental microbes, depending on how host-associated microbial communities are formed and maintained [16, 17]. In addition to these unique features of the host-associated microbes’ environment and ecology, host-associated microbial communities play fundamental roles in physiology, behavior and life history of their hosts given their key importance for essential functions like food digestion, ontogenetic development or protection against pathogens and parasites [14, 18–21]—the very traits that adapt hosts to their environment. Because of the fundamental roles that host-associated microbes play in animal physiology, behaviour and evolution, and associated coadaptation [22–24], associations between microbes and hosts can be tight [25, 26]. Hence, it is currently unclear whether the biogeographical structure of host-associated microbes resembles that often found for free-living microbes (“everything is everywhere”) or is determined by host traits. For example, currently unanswered questions include whether the assembly of host-associated microbial communities is driven by the environmental microbial communities or by host physiology and selection [27]. Therefore, studying geographical patterns of the host-associated microbial communities may contribute new perspectives to microbial biogeography.

Current literature on variation of host-associated microbes with geography is limited in scope and offers an equivocal picture [14]. Some single-species studies on various vertebrates show geographic variation in host-associated microbial communities [28–31], partly co-varying with geographic variation in host traits [29–31], whereas others do not find geographic variation in host-associated microbes [32–35]. These single-species studies are constrained by limited environmental variability as most hosts occur over only a small environmental range (e.g. [28, 34, 35]; but see [33]). A multi-species meta-analysis found important roles of both host species and sampling site in shaping bird gut microbiomes, with these factors ranked above others such as diet or captivity status [36]. Likewise, a recent interspecific study in European birds highlighted the relevance of geographic location in explaining gut microbial diversity [37]. However, another interspecific comparison found little evidence for a geographical effect on gut microbial communities of 59 Neotropical bird species [38]. Limitations for the interpretive power of these multi-species studies include (i) the small geographical scales (and the associated small environmental variability), and (ii) the confounded elements of variation in ecological niches and evolutionary historical trajectories due to the use of evolutionarily distantly-related hosts. This second limitation is particularly important given the proposed relevance of host evolutionary history in shaping host-microbe associations [23, 26]. Studies considering multiple host species covering large environmental variation, while sharing similar ecological niches and evolutionary histories, are required to shed more light on the role of geography in explaining variation in host-microbe associations.

An interesting model system to study biogeographical variation in host-associated microbial communities is the family of larks (*Alaudidae*) [39–46]. Larks comprise a group of globally-distributed, closely-related bird species with fundamentally similar ecologies (e.g. ground-nesting, ground-foraging, social life, diet), despite occurring in very different environments including tropical regions, desert areas and temperate zones [41, 47]. The use of closely-related hosts minimizes historical (co)evolutionary variation, which is an important factor that might affect the biogeography of host-associated microbial communities [10]. In an early study of the geographic co-variation between culturable free-living and host-associated microbes and the immune system of multiple lark species, Horrocks et al. [42] suggest that geographic location can play an important role in shaping host-microbe interactions. In addition, in a previous study using 16S rRNA gene amplicon sequencing Van Veelen et al. [48] show that sympatrically living woodlarks and skylarks do not differ in their gut microbial communities, providing no support for phylosymbiosis. Moreover, these authors suggest that the host-associated microbial communities of skylarks and woodlarks are largely shaped by host filtering of the environmental microbial communities.

Here we investigated how host-associated microbial communities vary with geography using a unique large geographical scale to study the variation in the gut microbial communities of nine closely related lark (*Alaudidae*) species from five different locations encompassing three biogeographical regions (desert, tropics, temperate zone). Using 16S rRNA gene amplicon sequencing, we first explored the influences of geographical location and host species in explaining differences in α-diversity of gut microbial communities of larks. Secondly, we analysed the geographic variation of dominant bacterial taxa and the co-occurrence of amplicon sequence variants (ASVs) in the lark-associated microbiota. Thirdly, we investigated the compositional similarity of bacterial communities (beta diversity) among locations and species. Finally, we asked to what extent geographical distances explain differences in gut microbial community characteristics among lark species, by comparing pairwise differences in the community composition of individual birds among and within locations.

## Methods

### Field sampling

We captured 125 individuals of nine lark species at five locations up to 6500 km apart. All locations were sampled during the breeding season for our study species at those sites. One sampling location (Aekingerzand, The Netherlands) was located in a temperate area and corresponds to the Eurasian biogeographical region. The two arid locations (Mahazat as-Sayd Protected Area and Taif, Saudi Arabia) belong to the Saharo-Arabian biogeographical region, while the other two sampling locations (Kinangop and Kedong, Kenya) were in the tropics within the African biogeographical region. Additional information on the specific environmental conditions of these locations can be found in [44, 49] and [50]. Details of species, sample sizes and year of sampling are provided in Table 1. We used the common technique of swabbing the cloaca of birds as proxy for gut microbial communities (e.g. [31, 48, 51]). We collected swabs by inserting the sterile swab approximately ~ 5 mm into the cloaca, and then gently rotated it for 10 s following previous recommendations [31, 51]. The swab was then placed in a sterile Eppendorf tube containing 250 ml of sucrose lysis buffer [31] that had been prepared under a sterilized fume hood (wiped clean with 70% ethanol and sterilized with a UV lamp for at least 5 min) and filtered through a sterile filter (0.2 µm) to remove any bacteria present. The swab was kept on ice in the field (< 8 h) and later frozen at -20 ºC the same day. Samples remained frozen until they were analysed in the lab.

**Table 1:** Sample size and geographic origin of bird species used in the study

Species	Latin name	n	latitude	longitude	Population	Country	Biogeographic region	Year
Skylark	*Alauda arvensis*	18	52º 56’ N	6º 18’ E	Aekingerzand	Netherlands	Temperate	2007
Woodlark	*Lullula arborea*	18	52º 56’ N	6º 18’ E	Aekingerzand	Netherlands	Temperate	2007
Hoopoe lark	*Alaemon alaudipes*	13	22º 20’ N	41º 44’ E	Mahazat as-Sayd	Saudi Arabia	Desert	2007
Bar-tailed lark	*Ammomanes cincturus*	3	22º 20’ N	41º 44’ E	Mahazat as-Sayd	Saudi Arabia	Desert	2007
Arabian lark	*Eremalauda eremodites*	18	22º 20’ N	41º 44’ E	Mahazat as-Sayd	Saudi Arabia	Desert	2007
Black-crowned sparrow-lark	*Eremopterix nigriceps*	14	21º 15’ N	40º 42’ E	Taif	Saudi Arabia	Desert	2007
Crested lark	*Galerida cristata*	4	21º 15’ N	40º 42’ E	Taif	Saudi Arabia	Desert	2007
Red-capped lark	*Calandrella cinerea*	8	0º 34’ S	36º 28’ E	Kinangop	Kenya	Tropics	2009
Rufous-naped lark	*Mirafra africana*	4	0º 34’ S	36º 28’ E	Kinangop	Kenya	Tropics	2009
Red-capped lark	*Calandrella cinerea*	23	0º 52’ S	36º 23’ E	Kedong	Kenya	Tropics	2009
Rufous-naped lark	*Mirafra africana*	2	0º 52’ S	36º 23’ E	Kedong	Kenya	Tropics	2009

### DNA extraction and 16S rRNA gene amplicon sequencing

We prepared cloacal swabs by aseptically peeling off the cotton from the stalk and placing this in an extraction vial (MoBio PowerSoil-htp 96 well DNA isolation kit, MoBio laboratories, Carlsbad, CA, USA), and performed DNA extractions as per manufacturer’s instructions. To improve cell disruption during three cycles of 60 s bead beating (Mixer Mill MM301, Retsch GmbH & Co, Germany) 0.25 g of 0.1 mm zirconia beads (BioSpec Products, Bartlesville, OK, USA) was added in the first step. The V4/V5 region of the 16S rRNA gene was amplified in triplicate using primers 515F and 926R at Argonne National Laboratory, IL, USA, following the Earth Microbiome Project protocol [52], followed by library preparation of pooled triplicates and 2 ⨉ 250 bp paired-end sequencing using V2 chemistry on an Illumina MiSeq platform. As part of a combined sequencing effort of multiple projects, in total 25 negative controls were included in amplification and sequencing. Two samples showed signs of PCR, but no reads passed quality filtering.

### Sequence data processing

We processed raw 16S rRNA sequence reads using the QIIME2 pipeline (v.2019.10; [53]). Sequence reads were demultiplexed, quality-filtered (median Phred ≥ 25) and primers trimmed. Briefly, we used the dada2-pluging [54] for sequence data error-correction and for inferring amplicon sequence variants (ASVs), which were dereplicated to construct the feature frequency table. We assigned taxonomic information to ASVs using a naïve Bayesian classifier [55] trained on a primer set-specific information extracted from the SILVA database (v.132) [56]. We then aligned the representative sequences using MAFFT [57], filtered gaps in the alignment and used the resulting alignment to construct a midpoint-rooted phylogeny with FastTree2 [58]. We then imported the QIIME2 output in R statistical software (v. 4.0.3) [59] for microbial community analyses using R packages *phyloseq* (v. 1.34.0) [60], *vegan* (v. 2.5–7).

We filtered ASVs assigned to Archaea, chloroplast and mitochondria, as well as ASVs that comprised > 0.01% of the total abundance. We retained 2,095,668 quality-filtered sequences covering 6178 ASVs, which comprised 99.98% of sequences in the unfiltered dataset. Rarefaction curves showed that ASV richness and Shannon diversity saturated at 4000 reads per sample for most samples (Fig. S1 [Additional file 1]). The coverage of our analysed samples ranged between 167 and 72,647 reads per sample.

### Statistical analyses—Comparing cloacal microbiota alpha diversity, co-occurrence and relative abundances of taxa among geographic locations and lark species

We estimated ASV richness (Chao1) and Shannon diversity of the cloacal microbiota after rarefying the ASV feature table to 4000 reads per sample (retaining 101 of 125 samples). Then we using linear mixed-effect models to analyse differences in (log-transformed) ASV richness and Shannon diversity among sampling locations with the packages *lme4* (v. 1.1–26) [61] and *lmerTest* (v. 3.1–3) [62]. We did not include phylogeny in the models because the lark species in this study are evenly distributed across the lark family tree [63] (Fig. S2 [Additional file 1]), and because phylogenetic corrections are only reliable with at least 20 species [64]. Instead, we used host species identity as a random factor in these analyses to account for the non-independence of individuals of the same lark species. We analysed differences between lark species with a model including host species as fixed effect. We used the *emmeans* package (v.1.7.5) [65] to explore pairwise differences between locations and host species based on Tukey post-hoc tests. We assessed the normality of residuals errors (Q-Q plots) and homoscedasticity of our models. We estimated the variance component of the host species random effect using the *specr* package (v.0.2.1) [66].

We then compared ASV co-occurrence among locations by means of Venn diagrams based on the rarefied data. To identify differentially abundant ASVs and phyla among the cloacal microbiota of larks at different geographic locations we performed analysis of composition of microbiomes with bias correction (*ANCOM-BC*; v.1.0.5) [67, 68] applying a critical false discovery rate (FDR)-corrected *q*-value of 0.05. Only samples with more than 1000 reads (n = 118 of 125) and ASVs with less than 90% zero-counts across samples were evaluated. We then visualised the centred log-ratio (clr) transformed counts of ASVs that differed significantly among locations in a heatmap with *ComplexHeatmap* (v.2.9.3) [69]. We calculated relative abundances of taxa at different taxonomic levels and visualized the averages per location in bar plots.

### Statistical analyses—Comparing community composition of lark cloacal microbiota among locations, lark species and in association with spatial distance

We assessed beta diversity based principal coordinates analysis (PCoA) of normalized ASV read counts of samples with at least 1500 reads (*n* = 118) to evaluate taxonomic (Bray–Curtis dissimilarities) and phylogenetic (weighted UniFrac) distances of cloacal microbiota among locations [70]. We performed constrained analysis using distance-based redundancy analysis with 999 permutations in the ‘adonis2’ function of the *vegan* package (v.2.5–7) [71–73] to test for compositional differences among geographic locations. While lark species are largely nested within location, this was not the case in the tropics. We dealt with the structure of our data by first testing if differences among locations affected community structure. In separate models, we tested differences among lark host species in terms of community composition. This approach provided qualitatively similar findings; we emphasize and discuss effects of geographic location, and for transparency and additional insights, include results from the host species model in the supplementary material [Additional file 1]. To explore pairwise differences between populations, we performed pairwise PERMANOVA with the ‘pairwise.perm.manova’ function [74]. We tested for the homogeneity of within-group dispersions among locations using the ‘betadisper’ function.

To explore associations between cloacal microbiota composition with spatial distance, we calculated the spatial distance among locations based on latitude and longitude of the geographic locations using the *sf* package [75]. Then, we plotted pairwise unweighted UniFrac distances among individuals for each geographic location as a function of geographic distance that represented their proximity in geographic location. We square-root transformed geographic distance to meet analysis criteria. To explore the average association pattern, evaluating if phylogenetic membership of microbiota of individuals geographically farther apart are more distinct, we compared a linear model with geographic distance to more complex models that additionally included either a quadratic or cubic term. Better model fit for the latter complex models may indicate that effects of location-specific environmental factors are stronger than simple isolation by linear distance.

## Results

### Richness and diversity of cloacal microbiota

**Fig. 1:**
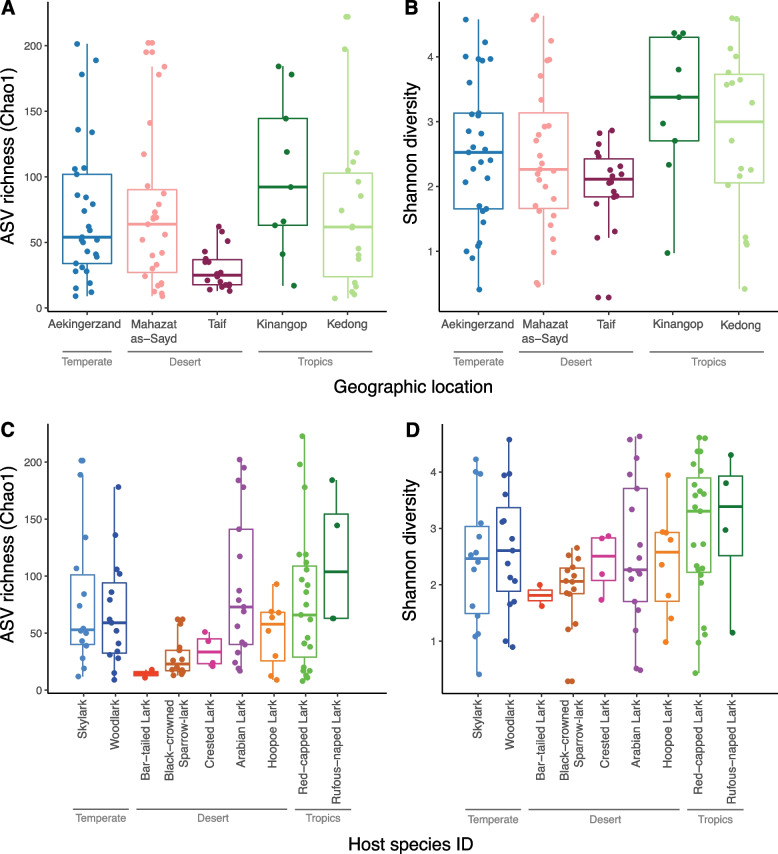
Alpha diversity of cloacal microbiota of larks. (A, C) Total estimated ASV richness (Chao1) and (B, D) Shannon diversity by geographic location and lark host species from temperate (blues), tropical (greens) and desert (other colors) habitats. Individual birds (closed circles) and their variation (box = (median, Inter Quartile Range (IQR)), whiskers (last value = <|1.5·IQR|)) are presented

The estimated ASV richness of lark cloacal microbiota did not differ between geographic locations (log Chao1: *F*_4, 2.5_ = 2.53, *P* = 0.33; Fig. 1A), but differed significantly between host species (*F*_8,92_ = 2.96, *P* = 0.005; Fig. 1B). Post hoc tests indicated significant differences only between Arabian larks and Black-crowned sparrow-larks; Arabian larks had 63 ASVs more in their cloacal microbiota than Black-crowned sparrow-larks (*t* = -3.37, Tukey *P*_adj_ = 0.03). Shannon diversity did not differ among geographic locations (*F*_4,96_ = 2.22, *P* = 0.07; Fig. 1C) or among host species (Shannon: *F*_8,92_ = 1.19, *P* = 0.31; Fig. 1D). Random effects for host species identity accounted for variance components of 16% for log-transformed Chao1 and 3% for Shannon diversity. Rank-abundance plots for each of the geographic locations produced with non-rarefied data showed that the cloacal microbiota of larks at desert locations were more skewed, Taif in particular (Fig. S3 [Additional file 1]), indicating dominance of a few relatively abundant ASVs that was less pronounced at other geographic locations.

### Co-occurrence patterns and relative taxon abundances of taxa

**Fig. 2:**
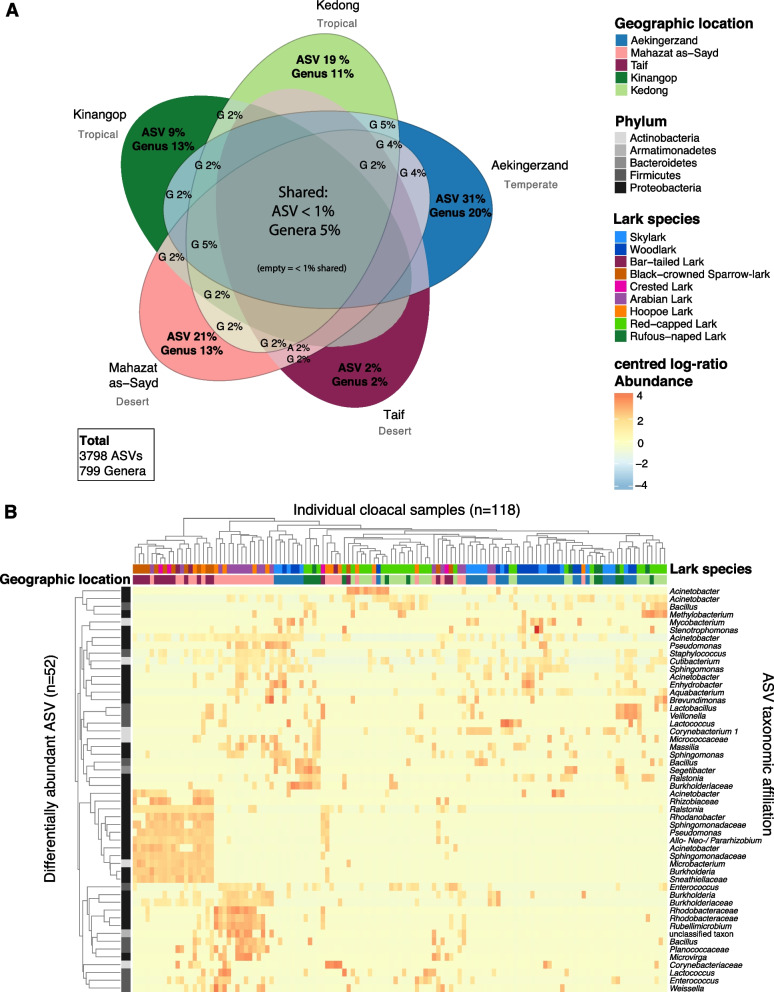
Bacterial co-occurrence and differential abundance in cloacal microbiota of larks from different geographic locations. (A) Shared and unique bacterial ASVs and genera in the cloacal microbiota of larks compared among geographic locations. For visual clarity genus is abbreviated as “G” and ASV as “A”, and overlapping fields without text represent < 1% shared ASVs and genera. Co-occurrences are based on rarefied data of 4000 reads per sample, and differentially abundant ASV (*n* = 52) were determined using ANCOM-BC on samples with more than 1000 reads and ASVs with at least 10% non-zero counts. (B) Distinct clustering of differentially abundant ASVs among locations

We found that 9 ASVs (< 1%) and 43 genera (5%) were shared among the cloacal microbiota of larks from all locations (Fig. 2A). Besides these low numbers of shared taxa, each location harboured a low to modest proportion of unique ASVs (2–31% of all ASVs, depending on location) and genera (2–20% of all genera), with Taif (desert) having the lowest proportions and Aekingerzand (temperate) the highest (Fig. 2A). Larks from the desert at Taif shared 4% of genera with larks at the nearby desert location Mahazat as-Sayd, and another 4% of genera with larks from the tropical dry grasslands at Kedong, Kenya (Fig. 2A). Considerably more shared genera were detected in comparisons involving the tropical locations at Kinangop and the temperate location Aekingerzand (Fig. 2A). Irrespective of the phylogenetic relationships among microbiota members, these co-occurrence patterns of bacterial ASVs and genera indicated that the cloacal microbiota of larks inhabiting distant and climatically distinct bioregions consist of a substantial proportion of unique taxa and to a lesser extent of shared taxa. Distinctiveness of cloacal microbiota of larks in the desert (Taif and Mahazat as-Sayd) was also manifested by the differential abundance analysis (Fig. 2B): The most prominent rectangular clusters of abundant ASVs corresponded with larks from the desert locations. Although less striking, also the temperate and tropical locations had characteristic abundant ASVs that uniquely occurred in each of these locations, including those affiliated to *Mycobacterium* and *Methylobacterium*, respectively.

**Fig. 3:**
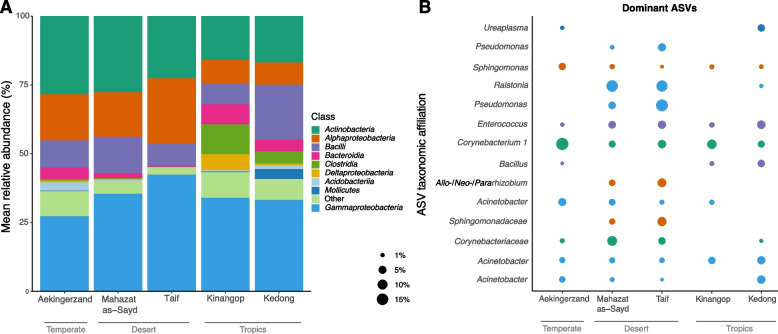
Bacterial community structure of lark cloacal microbiota across geographic locations. A) Relative abundances of bacterial classes and B) dominant ASVs in cloacal microbiota of larks inhabiting different geographic locations. Dominant ASVs were included when the mean relative abundance in cloacal microbiota of larks exceeded > 3% at one or more locations. Colours represent bacterial classes as in A)

Out of all 87 detected bacterial classes, we found that 8 classes belonging to five phyla (*Proteobacteria*, *Actinobacteria*, *Firmicutes*, *Bacteroidetes* and *Acidobacteria*) dominated larks’ cloacal gut microbiome (location: Fig. 3A; species: Fig. S4 [Additional file 1]). Overall, *Proteobacteria* was the most dominant phylum in larks at all locations (> 45%). *Actinobacteria* was the second most abundant class, but not in tropical African larks where *Bacilli* and *Clostridia* (phylum *Firmicutes*) and *Bacteroidia* (phylum *Bacteroidetes*) were subdominant phyla. The relative abundances of 14 phyla differed significantly among locations, with the exception of the two dominant phyla (ANCOM-BC, FDR *q* < 0.05; Table S1 [Additional file 1]). The nine classes that covered more than 85% of reads showed that in the tropical locations more classes including *Clostridia*, *Deltaproteobacteria*, and *Mollicutes* were part of the dominant taxa, compared to the desert and temperate locations (Fig. 3A).

Subsequent more detailed assessment of the dominant taxa across all lark samples, revealed 14 abundant ASVs in lark cloacal microbiota (Fig. 3B). These ASVs include bacteria of the genus *Corynebacterium 1* which was dominant at temperate Aekingerzand, and multiple abundant taxa at the desert locations, most notably *Ralstonia* and *Pseudomonas* ASVs, which is in line with the steep ascending curve observed in a rank-abundance plot of desert Taif (Fig. S3 [Additional file 1]). ASVs belonging to *Acinetobacter*, *Bacillus* and *Ureaplasma* had the highest relative abundances in tropical larks. The prominent presence of *Mollicutes* at Kedong (Fig. 3A) resulted from an individual Rufous-naped lark with extreme abundance of this bacterial taxon, which is often associated to pathogenic mycoplasma (Class *Mollicutes*, genus *Ureaplasma*), and was found in lower abundance in three other Rufous-naped larks and a Skylark.

### Community composition among geographic locations and lark species

**Fig. 4:**
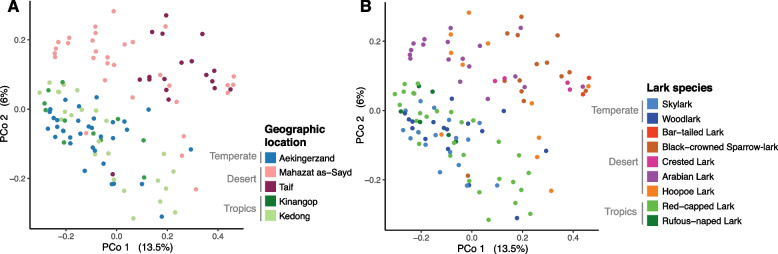
Cloacal microbiota composition of larks. Ordination of principal coordinates based on unweighted UniFrac distances between cloacal microbiota of larks, coloured by (A) geographic location and (B) host species. Clustering patterns in ordinations show the distinct phylogenetic memberships of bacterial lineages in the microbiota of desert larks compared to temperate and tropical larks

Analysis of beta diversity based on unweighted UniFrac distances revealed that the phylogenetic membership of cloacal microbiota differed among geographic locations (PERMANOVA, pseudo-*F*_4,117_ = 4.17, R^2^ = 0.13, *P* = 0.001; Fig. 4A). Clustering patterns in the ordination plots demonstrated that the cloacal microbiota of larks in the deserts at Mahazat as-Sayd and Taif had a different phylogenetic structure than tropical and temperate larks (Fig. 4A). Pairwise PERMANOVA tests revealed significant differences among all locations (0.001 < *P* < 0.003), except between tropical Kedong and tropical Kinangop (*P* = 0.07). Besides geographic location, an additional 5% of variation in phylogenetic membership could be explained by lark host species (db-RDA, pseudo-*F*_5, 117_ = 1.33, *P* = 0.005). Distinctive taxonomic composition of desert lark microbiota from Taif was also observed based on PCoA of Bray–Curtis dissimilarities (Fig. S5 [Additional file 1]), but less pronounced than for phylogenetic membership (Fig. 4). Because of significant beta-dispersion (within-group variance) between geographic locations (*F*_4,113_ = 8.43, *P* < 0.001; Fig. 4A), interpretations were mainly based on the clustering patterns. The clustering of samples by geographic location while accounting for ASV relative abundances (Bray–Curtis) was weaker than clustering based on lineage occurrences (unweighted UniFrac distances).

### Association between cloacal microbiota composition and geographic distance

**Fig. 5:**
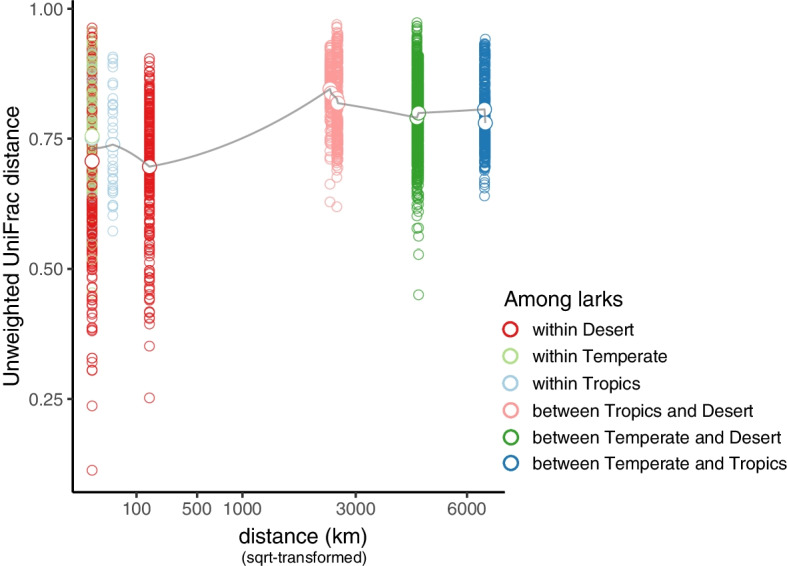
Cloacal microbiota compositional variation in larks with geographic distance. Pairwise unweighted UniFrac distances between cloacal microbiota larks (*n* = 118) are separated by the geographic distance (square-root transformed) between individuals of each pair. Pairs are coloured by the unique combination of biogeographic regions of their origin. Mean unweighted UniFrac distances of each among-location comparison are depicted by large circles and connected by a grey line to highlight the non-linear pattern of cloacal microbiota similarity with distance

Pairwise unweighted UniFrac distances among all pairs of larks were significantly explained by geographic distance, but this relationship was non-linear (Fig. 5). Comparing models to predict pairwise unweighted UniFrac by geographic distance revealed that a polynomial models fit the data best (sqrt_distance: adj. r^2^ = 0.07, AICc = 5860; adding sqrt_distance^2^: adj. r^2^ = 0.08, delta AICc = -10; adding sqrt_distance^3^ = adj. r^2^ = 0.08, delta AICc = -12).

## Discussion

This study is the first to explore large-scale patterns of geographic variation in cloacal microbial communities of free-living birds using a multispecies comparison and across three biogeographical regions. Our results reveal substantial geographical structure in bird-associated microbial communities, despite the overall relatively constant environment provided by different birds’ bodies, and contrary to the “everything is everywhere” hypothesis. This geographical structure in lark cloacal gut microbial communities is evident with respect to taxon co-occurrence patterns (Fig. 2A), evenness (Fig. S3 [Additional file 1]), dominant taxonomic groups and their relative abundances (Fig. 3) as well as in community composition (Fig. 4), but not present in patterns of ASV richness and Shannon diversity (Fig. 1). In addition, we found that geographic distance was associated with pairwise unweighted UniFrac distances, in a polynomial way. This finding suggested that not only geographic distance is important to explain variation in cloacal gut microbiota, but also location-specific environmental and climatic conditions (Fig. 5). Our geographic patterns of host-associated microbial communities resemble biogeographic patterns found in higher taxonomic groups (e.g. vertebrates) including different community structure in deserts compared to tropical areas, and environment-dependent adaptations of host physiological and life-history traits [1–4, 46]. The geographic differences and commonalities raise questions about the role of environmental microbial communities as source for host-associated microbiota, about codiversification of microbial lineages with hosts, and about the potentially functional relationships between host-associated microbes and host-adaptive traits.

Our finding that host-associated microbial community structure varied with geography despite the generally relatively constant environment provided by larks’ bodies (Fig. 2, Fig. 3, Fig. S3 [Additional file 1]), raises questions about the different selective forces that determine the distribution of free-living and host-associated microbial communities, and about the connections between free-living and host-associated microbial communities. This finding is relevant in the current debate on whether all life forms are equally affected by biogeography [9, 10] and has important implications for the evolutionary processes shaping both macro and microorganisms [14, 20]. Previous local or regional studies focused on host-associated microbial communities have found that geography explained several α-diversity metrics in microbiota of birds [31, 35] and other vertebrates [28, 30, 33]. Expanding on this earlier work, our study adds for the first time at a large biogeographical scale evidence that host-associated microbes do not fit the “everything is everywhere” hypothesis. We hypothesize that some causes used to explain the “everything is everywhere” hypothesis for free-living microbes (e.g. high dispersal abilities [10, 11, 76]) could be altered due to the association with hosts. For example, processes such as host selection of host-associated communities by filtering from the pool of environmental microorganisms, could be (at least partially) responsible for cloacal gut microbial assemblages. Previous studies that show that culturable free-living and host-associated bacteria of larks are less abundant in the desert compared to less arid areas [42], and that the environmental microbial communities play a large role in the acquisition of gut microbes in two temperate larks [48], are also in line with the hypothesis that gut microbial assemblages are impacted by free-living environmental bacterial communities. Multi-species, large-scale studies that include pairwise comparisons of both free-living and host-associated microbes from the same study sites would be a first step towards further testing the hypothesis that host-associated microbiota are (partially) selected from free-living environmental communities.

Our results on beta diversity, notably the geographic variation revealed by the unweighted UniFrac analysis (Fig. 4), also shed light on the processes that might shape geographical differences in lark-associated microbial assemblages, particularly co-diversification of microbial lineages with hosts and uptake of host-associated microbes from the environmental pool. The unweighted UniFrac analyses highlight the distinctiveness of desert locations (Taif and Mahazat) regarding phylogenetic community composition (Fig. 4). The phylogenetic differences are partially illustrated by the dominant ASVs for each location (Fig. 3B). These results potentially indicate different co-evolutionary historical processes of host species at different locations or, alternatively, phylogenetically different environmental bacterial pools at different locations. Overall, the geographic effects in our unweighted UniFrac (Fig. 4) and Bray–Curtis analyses (Fig. S5 [Additional file 1]) match another recent multi-species comparative analysis of gut microbial assemblages in a group of temperate-zone phylogenetically-distant birds, as well as partially match (Bray–Curtis results) studies with other birds [29, 38] and vertebrates [28]. This gives support for the generality of our findings. However, additional multi-species comparative studies controlling for the co-evolutionary history of hosts (e.g. restricting to closely-related species, or taking into account phylogenetic relationships among hosts), using large-scale geographic comparisons, and potentially using other vertebrate host taxa or host-associated materials would be required before drawing further conclusions on the contribution of co-evolutionary historical processes in explaining geographic variation in host-associated microbial communities.

In addition to demonstrating that host-associated microbes do not follow a distribution compatible with the “everything is everywhere” hypothesis, a key finding of our study is that host-associated microbes can follow large-scale macro-ecological patterns. One such well-known pattern is that of lower species richness in arid areas [2] compared to tropical regions [3, 4]. In our study, the main difference in cloacal gut community structure was detected between lark species from the two desert sites and the other lark species. In addition, like in macro-ecological patterns, we found that with larger geographic distance the host-associated microbial community compositions as described by pairwise unweighted UniFrac distances diverged more (Fig. 5). Although the shape of the relationship is non-linear, it provides additional evidence that for host-associated microbes the hypothesis that “everything is everywhere” is not supported. The non-linear pattern suggests that large-scale differences among biomes, such as environmental microbial communities, could cascade through into bird microbiota. Differences in locally adapted microbiota associated with vegetation and insect communities, which evolved to thrive in their specific climatic conditions, potentially horizontally transfer to bird microbiota through diet of foraging birds. Investigating why biogeographic rules might affect host-associated microorganisms similarly to macro-organisms and differently from free-living microbes is essential for understanding the processes that shape microbial assemblages [14]. Based on the differences with free-living microbes, it is possible that the host is playing an intermediate role, either through co-diversification of host and specific microbes or through functional links of specific microbes with host adaptive traits, favouring the influence of large-scale biogeographic patterns in microbes.

Geographic variation in host-associated microbial communities could result if these host-associated microbial communities have functional relationships with adaptive traits of hosts, such as adjustments in physiology and life history to live in different environments [19, 20]. Previous investigations of physiologies and life histories of the lark species from the same locations as used in this study have highlighted differences among desert, tropical and temperate zone larks. Desert larks have lower immune response, lower growth rates, smaller and fewer clutches per year, as well as lower basal metabolic rate compared with temperate larks, while they also differ from tropical larks with respect to immune function and reproductive strategy [40–44]. Interestingly, our results also highlight the uniqueness of the cloacal gut microbial communities of desert larks. For instance, lark gut microbial communities in the desert were dominated by a low number of relatively abundant ASVs compared with the other geographic locations (Fig. S3 [Additional file 1]). These results in addition to those regarding dominant bacterial groups at different taxonomic levels and differential abundances (Fig. 2B, Fig. 3B and Table S1 [Additional file 1]) demonstrate the distinctiveness of cloacal gut microbial communities of lark species at the two desert locations, compared with the temperate and tropical sites. Furthermore, our beta diversity analysis indicates that the geographic differences in gut microbiome composition of larks are mainly due to bacterial communities of desert larks (Fig. 4A). These pieces of evidence, together with previous studies on the physiology of larks [40–44, 77] illustrate the co-variation between gut microbes and the physiological and life-history traits that adapt hosts to their environment. Whether these lark-associated bacteria provide their hosts with specific functions or are simply the by-product of unique environmental ASVs incorporated into their gastrointestinal tract by different processes (e.g. via ingestion with food [78]) remains unknown. However, given the importance of gut microbes for some key functions of their hosts [18–21] including those previously analyzed for larks (e.g. immune function, metabolism and growth; [42–44]), we hypothesize that there may be functional associations between the cloacal gut microbes and the adaptations of larks to their respective environments [44]. To investigate this intriguing possibility, additional studies are required to further explore these potential functional relationships and to what extent gut microbes could contribute to the adaptive values of these host traits, which is an important gap in current microbiology and animal ecology [19]. In general, future studies should confirm the generality of our findings by also including different animals and different body parts, paying special attention to integrate hosts from arid areas into their comparisons. Overall, our study provides a novel example of the importance of integrating host-associated microbes into the field of microbial biogeography in order to advance not only our understanding on key biogeographic questions but also on the evolution of host-microbe interactions.

A limitation of this work includes the difficulty to distinguish microbiota members from contaminant taxa in low biomass samples. A priori filtering of potential contaminant taxa is a preferred universal solution for accurate microbiota profiling [79, 80], but is less fitting when studying animals interacting with soils. A notable complicating aspect is that designated contaminants are often abundant and globally widespread soil bacteria [81]. A global meta-analysis including human, great ape and insect microbiota demonstrated that these soil bacteria (i.e. common contaminants [79, 80]) are ecologically significant in the assembly of animal microbiota [81]. Many of the designated contaminant taxa can be dominant microbiota members in birds, their insect prey or avian parasites (e.g., *Pseudomonas*, *Ralstonia*, *Mycobacterium*, *Methylobacterium* [82–85]). Within *Alaudidae* (larks), direct microbiota comparisons between individual birds and their soil and nest microbiota revealed marked occurrence of soil and nest bacteria in cloacal and feather microbiota [48]. Additional experimental evidence for this sourcing of cloacal microbiota with soil bacteria in captive zebra finches was also reported [16]. Thus, in microbiota studies of birds (or other animals) that predominantly scavenge topsoil for plant seed and other food items, confounding effects of soil contaminants will remain difficult to distinguish from taxa that have evolved functional roles in both soil and host ecosystems. Hence, precautions are crucial to prevent contamination particularly when only small biomass samples can be collected, such as cloacal swabs. We argue that post hoc removal of contaminant taxa may inadvertently alter true biological patterns of animal microbiota profiles, and that extra care in sampling procedures is warranted, while DNA concentration data and sequencing serial dilutions of samples could help detect true contaminant presence in future studies [79, 86].

## Supplementary Information

**Additional file 1.**

**Additional file 2.**

**Additional file 3.**

## Data Availability

The QIIME2 workflow script as well as the R markdown file created for study are available as supplementary material (Additional file 2 and 3). The analyses in R can be reproduced from GitHub: https://github.com/pietervanveelen/biogeography_lark_microbiota. Sequence data are available at the European Nucleotide Archive (ENA) under project number PRJEB51018.
