# Supplementary material for: Cloacal microbiota are biogeographically structured in larks from desert, tropical and temperate areas
Source: BMC Microbiol. 2023 Feb 11;23:40. doi: 10.1186/s12866-023-02768-2 (PMC9921332; doi:10.1186/s12866-023-02768-2)
Supplement: Supplementary file 1 — Additional file 1. [file 12866_2023_2768_MOESM1_ESM.pdf]

Additional file 1

This document provides supplementary figures and tables accompanying the article **Cloacal microbiota are biogeographically structured in larks from desert, tropical and temperate areas**
van Veelen HPJ, Ibáñez-Álamo J-D, Horrocks NPC, Hegemann A, Ndithia NK, Shobrak M, Tieleman BI.

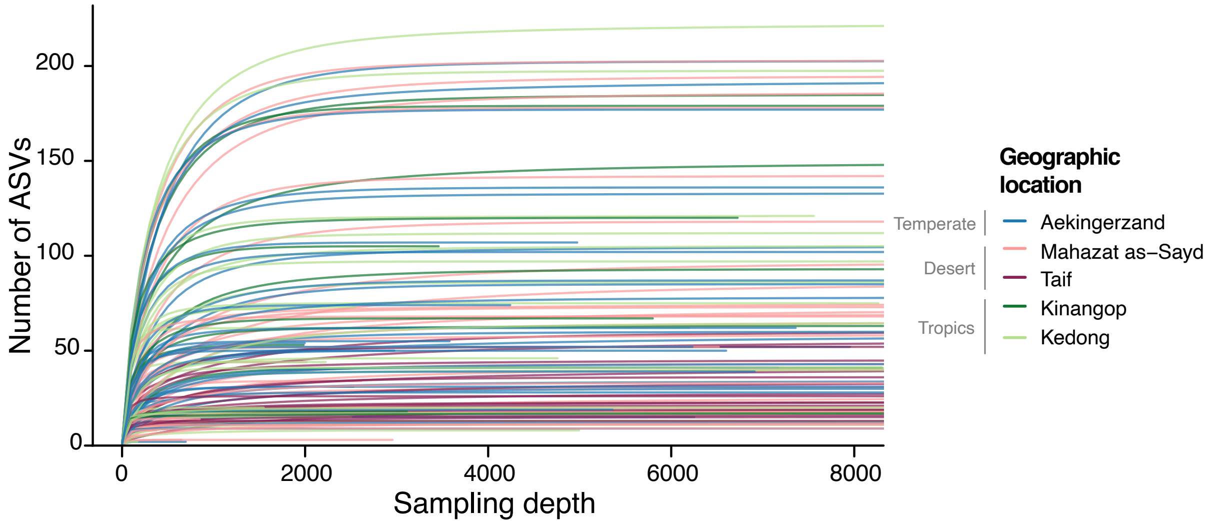

Figure S1. Rarefaction curves of ASV richness. Sampling depth is expressed as the number of reads per sample.

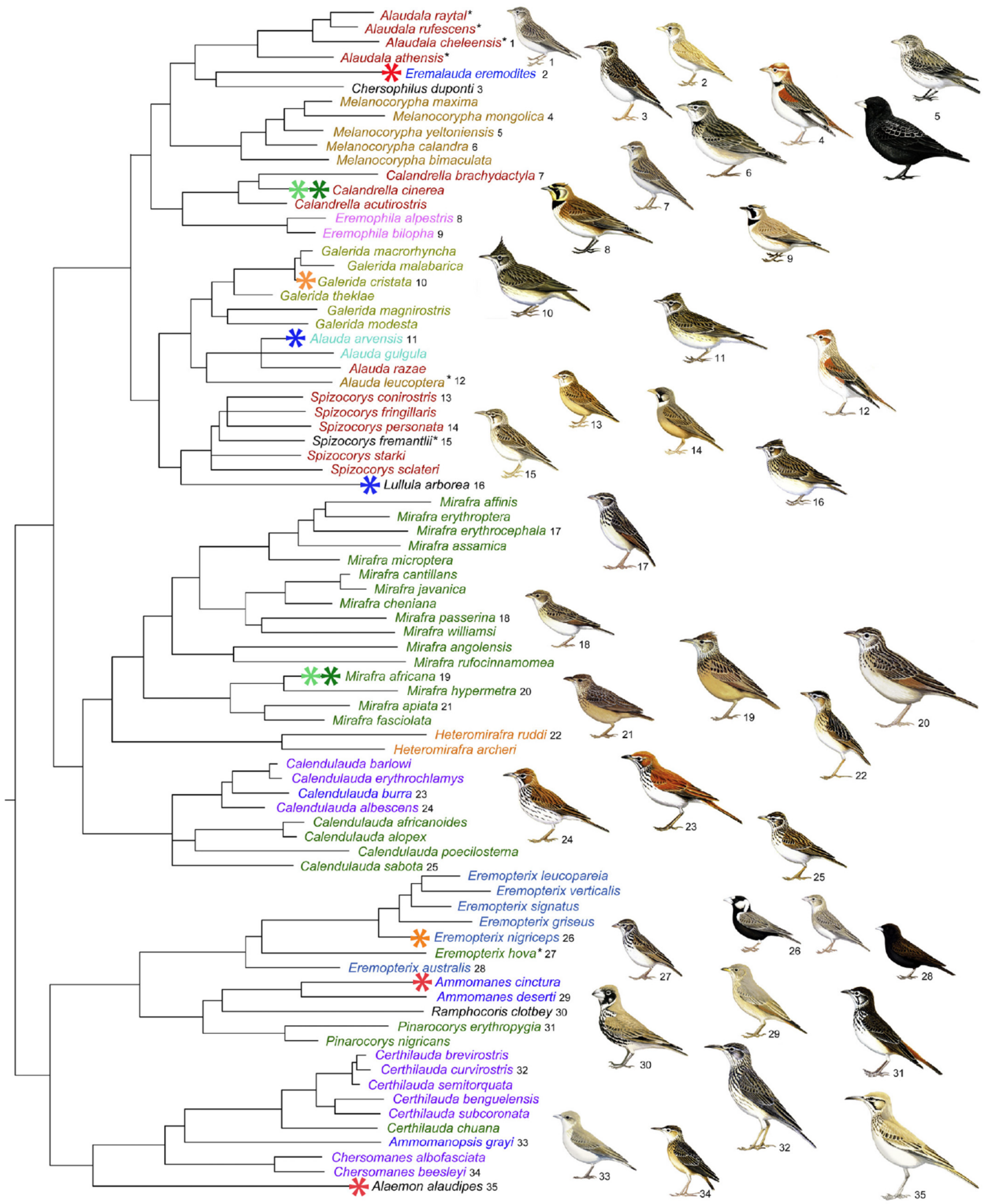

Figure S2. Phylogenetic relationship of the *Alaudidae* family. With permission from the authors, reproduced and adjusted from Alström et al., 2013 [1]**.** Lark species included in this study are marked with an asterisk (blue: Aekingerzand (temperate), red: Mahazat as-Sayd (desert), orange: Taif (desert), light green: Kedong (tropics), dark green: Kinangop (tropics)).

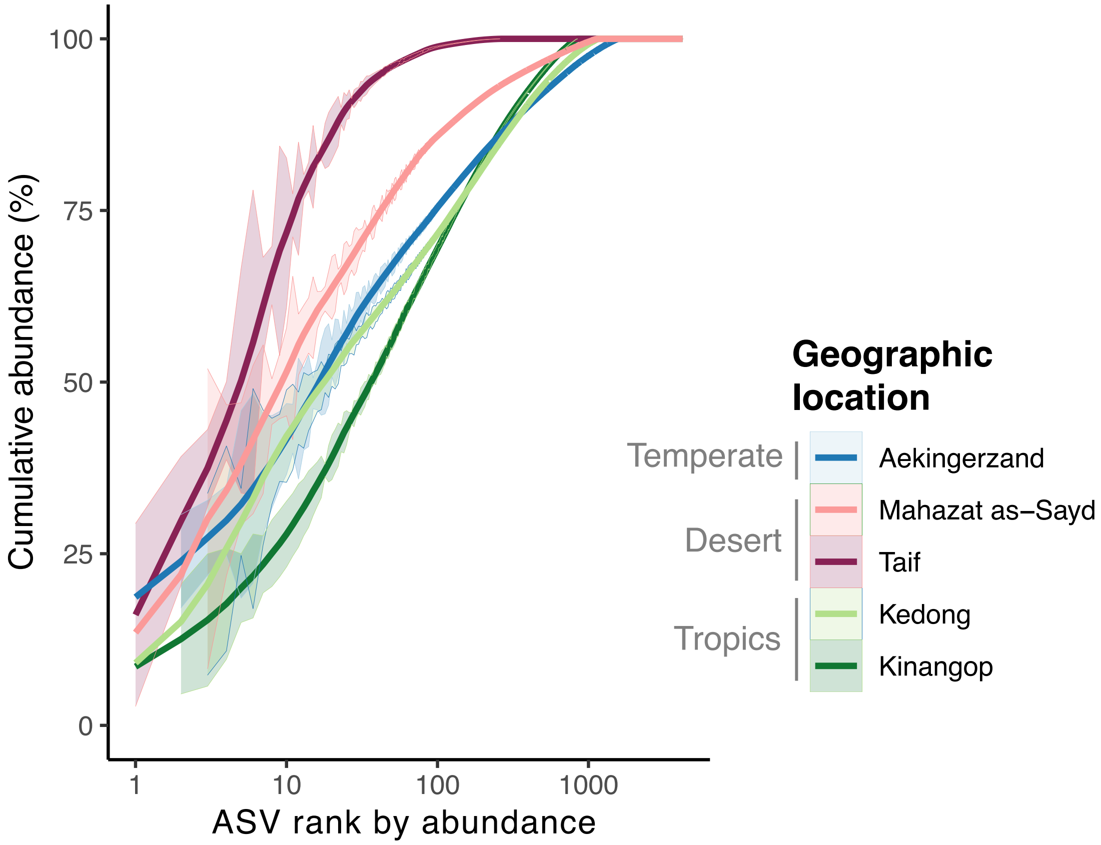

Figure S3. Rank-abundance curves of cloacal microbiota of larks. The two steepest increasing curves belong to desert sites Taif and Mahazat as-Sayd and show lower evenness at these locations than in the tropical sites (Kedong and Kinangop) and the temperate site (Aekingerzand), i.e. cloacal microbiota were more dominated by a few taxa at desert sites.

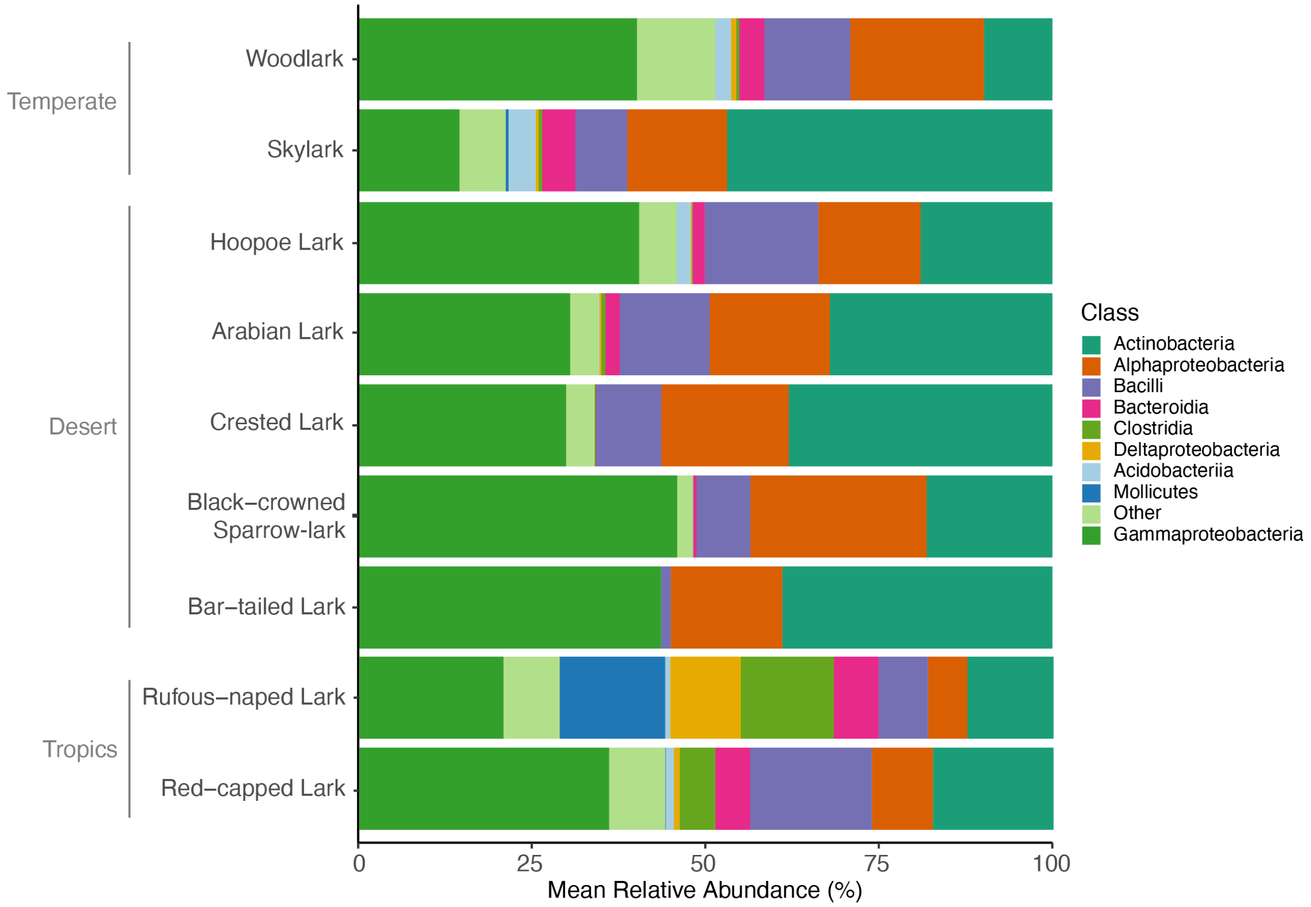

Figure S4 Relative abundances of bacterial classes in cloacal microbiota of nine lark host species.

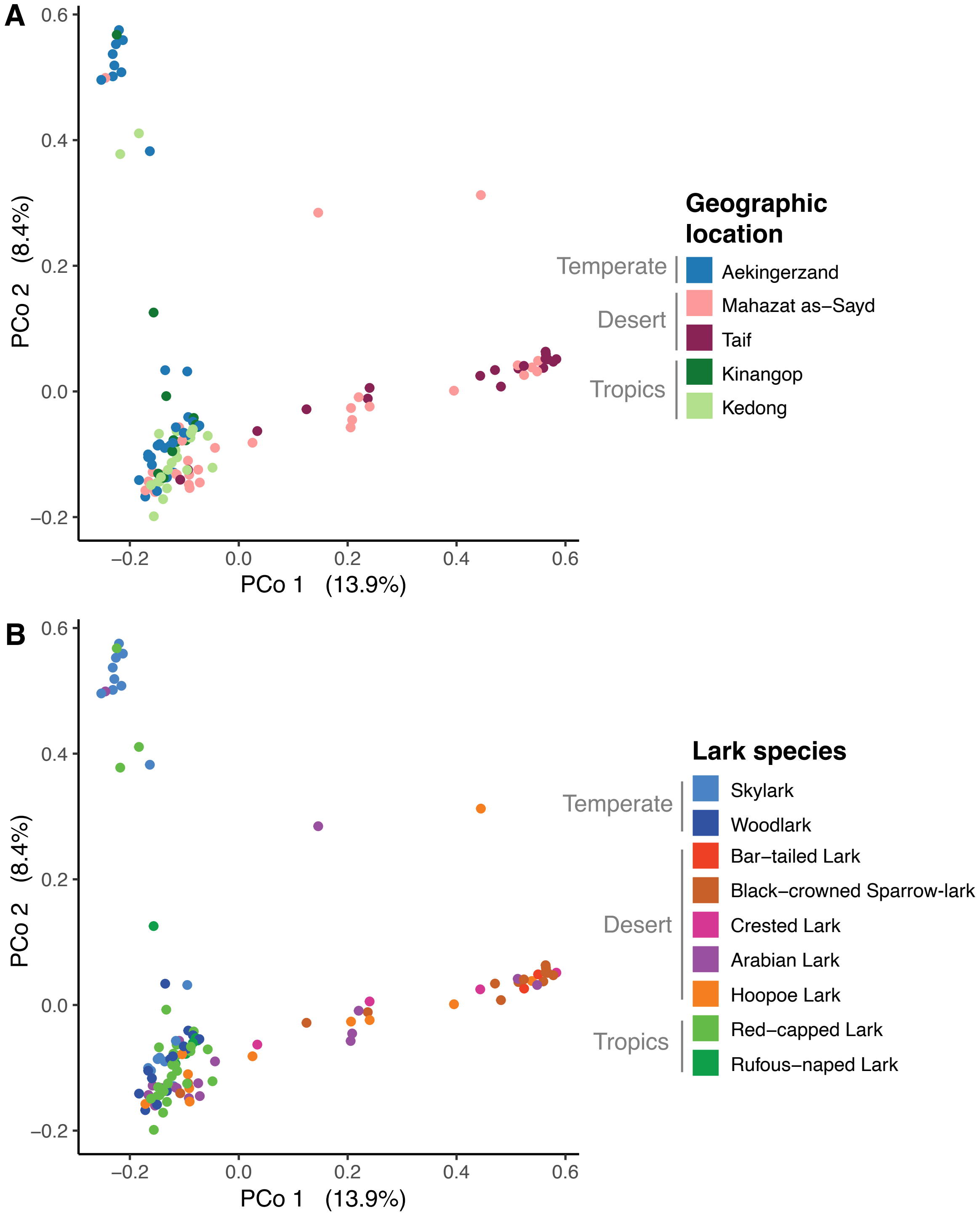

Figure S5. Cloacal microbiota composition of larks. Ordination of principal coordinates based on Bray-Curtis dissimilarities between cloacal microbiota of larks at (A) five geographic locations and by (B) host species.

Table S1 Bacterial phyla identified as differentially abundant among geographic locations. ANCOM-BC test statistic W-values and associated p-values and FDR-corrected q-values are presented.

Phylum	W	P value	q value
Chloroflexi	126.74	<0.001	<0.001
Acidobacteria	96.08	<0.001	<0.001
Armatimonadetes	79.68	<0.001	<0.001
Bacteroidetes	70.71	<0.001	<0.001
Thaumarchaeota	47.05	<0.001	<0.001
Gemmatimonadetes	41.55	<0.001	<0.001
Planctomycetes	36.45	<0.001	<0.001
WPS-2	35.77	<0.001	<0.001
Deinococcus-Thermus	35.06	<0.001	<0.001
Firmicutes	25.09	<0.001	<0.001
Euryarchaeota	25.00	<0.001	<0.001
Verrucomicrobia	14.59	<0.001	<0.001
Proteobacteria	14.58	0.01	0.06
Cyanobacteria	14.06	<0.001	<0.001
Actinobacteria	4.71	0.64	1
Epsilonbacteraeota	2.82	<0.001	<0.001

References

1. Alström P, Barnes KN, Olsson U, Barker FK, Bloomer P, Khan AA, et al. Multilocus phylogeny of the avian family Alaudidae (larks) reveals complex morphological evolution, non-monophyletic genera and hidden species diversity. Mol Phylogenet Evol. 2013;69:1043–56.
