# Supplementary material for: Cloacal microbiota are biogeographically structured in larks from desert, tropical and temperate areas
Source: BMC Microbiol. 2023 Feb 11;23:40. doi: 10.1186/s12866-023-02768-2 (PMC9921332; doi:10.1186/s12866-023-02768-2)
Supplement: Supplementary file 2 — Additional file 2. [file 12866_2023_2768_MOESM2_ESM.docx]

#!/bin/bash

#SBATCH --job-name=BASH_ZF_PV

#SBATCH --nodes=1

#SBATCH --ntasks-per-node=1

#SBATCH --cpus-per-task=16

source activate /export2/home/pvee/miniconda3/envs/qiime2-2019.10

cd /export2/home/pvee/RUG_data_zf/

# rename and organise input files

mv ./*run1/*I1* ./emp_paired_run1/barcodes.fastq.gz

mv ./*run2/*I1* ./emp_paired_run2/barcodes.fastq.gz

mv ./*run3/*I1* ./emp_paired_run3/barcodes.fastq.gz

mv ./*run4/*I1* ./emp_paired_run4/barcodes.fastq.gz

mv ./*run1/*R1* ./emp_paired_run1/forward.fastq.gz

mv ./*run2/*R1* ./emp_paired_run2/forward.fastq.gz

mv ./*run3/*R1* ./emp_paired_run3/forward.fastq.gz

mv ./*run4/*R1* ./emp_paired_run4/forward.fastq.gz

mv ./*run1/*R2* ./emp_paired_run1/reverse.fastq.gz

mv ./*run2/*R2* ./emp_paired_run2/reverse.fastq.gz

mv ./*run3/*R2* ./emp_paired_run3/reverse.fastq.gz

mv ./*run4/*R2* ./emp_paired_run4/reverse.fastq.gz

# import runs separately

qiime tools import \

--type EMPPairedEndSequences \

--input-path emp_paired_run1 \

--output-path emp_paired_run1.qza

qiime tools import \

--type EMPPairedEndSequences \

--input-path emp_paired_run2 \

--output-path emp_paired_run2.qza

qiime tools import \

--type EMPPairedEndSequences \

--input-path emp_paired_run3 \

--output-path emp_paired_run3.qza

qiime tools import \

--type EMPPairedEndSequences \

--input-path emp_paired_run4 \

--output-path emp_paired_run4.qza

# demultiplex each data set

runlist='run2 run3 run4' # run1 takes out, because of completed test run on run1 data

for run in $runlist;

do

qiime demux emp-paired \

--m-barcodes-file /export2/home/pvee/RUG_data_zf/mapping_files/all_run_samples/mapping_$run.txt \

--m-barcodes-column BarcodeSequence \

--p-no-golay-error-correction \

--i-seqs emp_paired_$run.qza \

--output-dir demux_$run

done

sleep 2

# run dada2 on each data set

# set maxEE on 4 an 4 for for and rev read

# trim lengths at 240 and 200 for for and rev reads

runlist='run1 run2 run3 run4'

for run in $runlist;

do

qiime dada2 denoise-paired \

--i-demultiplexed-seqs /export2/home/pvee/RUG_data_zf/demux_$run/per_sample_sequences.qza \

--p-trim-left-f 10 \

--p-trim-left-r 10 \

--p-trunc-len-f 240 \

--p-trunc-len-r 200 \

--p-max-ee-f 4 \

--p-max-ee-r 4 \

--output-dir dada2_${run}_ee44 \

--p-n-threads 16

done

sleep 2

# merge output data

qiime feature-table merge \

--i-tables /export2/home/pvee/RUG_data_zf/dada2_run1_ee44/table.qza \

--i-tables /export2/home/pvee/RUG_data_zf/dada2_run2_ee44/table.qza \

--i-tables /export2/home/pvee/RUG_data_zf/dada2_run3_ee44/table.qza \

--i-tables /export2/home/pvee/RUG_data_zf/dada2_run4_ee44/table.qza \

--o-merged-table merged_table.qza

qiime feature-table merge-seqs \

--i-data /export2/home/pvee/RUG_data_zf/dada2_run1_ee44/representative_sequences.qza \

--i-data /export2/home/pvee/RUG_data_zf/dada2_run2_ee44/representative_sequences.qza \

--i-data /export2/home/pvee/RUG_data_zf/dada2_run3_ee44/representative_sequences.qza \

--i-data /export2/home/pvee/RUG_data_zf/dada2_run4_ee44/representative_sequences.qza \

--o-merged-data merged_rep-seqs.qza

sleep 2

# make alignment

qiime alignment mafft \

--i-sequences merged_rep-seqs.qza \

--o-alignment aligned_merged_rep-seqs.qza \

--p-n-threads 16

sleep 2

qiime alignment mask \

--i-alignment aligned_merged_rep-seqs.qza \

--o-masked-alignment masked_aligned_merged_rep-seqs.qza

# create phylogeny

qiime phylogeny fasttree \

--i-alignment masked_aligned_merged_rep-seqs.qza \

--o-tree unrooted_tree.qza \

--p-n-threads 16

qiime phylogeny midpoint-root \

--i-tree unrooted_tree.qza \

--o-rooted-tree rooted_tree.qza

sleep 2

# assign taxonomy

qiime feature-classifier classify-sklearn \

--i-classifier NB_classifier_SILVA_132_99_16S_515F-926R_QIIME2-2019.10.qza \

--i-reads merged_rep-seqs.qza \

--o-classification taxonomy_NB_classifier_SILVA_132_99_16S_515F-926R_QIIME2-2019.10.qza \

--p-n-jobs 16

sleep 2

source deactivate
